# Scientific Cooperation and the Co-production of Scientific Outcomes for Physical Activity Promotion: Results From a Transdisciplinary Research Consortium

**DOI:** 10.3389/fpubh.2021.604855

**Published:** 2021-06-11

**Authors:** Susanne Ferschl, Maike Till, Karim Abu-Omar, Klaus Pfeifer, Peter Gelius

**Affiliations:** Department of Sport Science and Sport, Friedrich-Alexander-University of Erlangen-Nuremberg, Erlangen, Germany

**Keywords:** cooperation of science teams, The Science of Team Science, transdisciplinary research consortium, physical activity promotion, collaborative readiness, collaborative products

## Abstract

**Background:** To tackle complex societal challenges such as the high prevalence of physical inactivity, research funding is increasingly channeled toward cross-disciplinary research consortia. This study focused on exchange and cooperation (E&C) among the scientists of a 5-year transdisciplinary research initiative in Germany. Researchers' perceptions of E&C were combined with numbers of collaborative products during the project's life to make the developments of E&C and the quality of collaborative products visible.

**Methods:** We applied a mixed-methods design including a qualitative content analysis of pre-interviews, focus-group interviews, and documents as well as a quantitative analysis of research (scientific publications, books, conference participations) and training outcomes (supervised bachelor's, master's, and Ph.D. theses). Inductive and deductive approaches were combined to analyze factors of collaborative readiness and to identify perceptions of E&C among project teams. Based on Hall et al.'s “Conceptual Model for Evaluation of Collaborative Initiatives,” the project period was separated into phases of “collaborative readiness,” “collaborative capacity,” and “collaborative products.”

**Results:** Our findings revealed a discrepancy between the objectively assessed concepts of collaborative readiness and researchers' reported perceptions of E&C during the early project stage. A set of E&C hindering factors identified during the initial project phase remained present until the final project stage. Further, E&C among scientists increased over time, as reflected by researchers' perceptions. Reports of scientists also showed that outcomes were co-produced at the final project stage for the first time, while knowledge integration had not yet been achieved. Generally, the number of collaborative products (particularly scientific publications) also substantially increased over time. E&C was supported and promoted by the efforts of the coordinating sub-project.

**Conclusion:** Scientific E&C is a learning process and needs time to develop. A participatory research approach taking into account the perspectives on and requirements for E&C during the project's design might lay the ground for suitable, supportive, and transparent conditions for effective and successful E&C. Despite their time- and resource-consuming nature, cross-disciplinary research initiatives provide a fertile context in which to generate new solutions for pressing societal issues given that long-term funding and the establishment of an overarching coordination organ is assured.

## Introduction

Complex societal problems such as climate change as well as health issues such as tobacco use or physical inactivity are increasingly addressed through the cooperation and knowledge integration of different scientific disciplines (1–3). Although cross-disciplinary (e.g., inter-, multi-, or transdisciplinary) exchange and cooperation (E&C) is a promising basis for scientific and societal advancements (4), it is complex and entails challenges at the individual, team, conceptual, institutional, and coordination levels (5, 6). Specifically, coordination, collaborative problem-solving, and the production of outcomes in initiatives with researchers located at different universities require time to develop (7–10).

In the context of academia, E&C is characterized by very specific conditions, such as the importance of a sound reputation or the competition for jobs, grants, and publication opportunities (11). Bozeman and Boardman have defined scientific collaboration as the “social processes whereby human beings pool their experience, knowledge and social skills with the objective of producing new knowledge, including knowledge as embedded in technology” (12). It has been shown that researchers were motivated to participate in research collaborations due to access to expertise and resources, recognition and reward, higher productivity, and the learning of new skills (13, 14). In contrast, the risks of collaboration in research include the critical assignment of credits and high coordination costs (13).

For cross-disciplinary E&C resulting in knowledge integration, several requirements have been identified in the literature. New methods and concepts need to be developed systematically and appropriate communication tools and institutional arrangements established (15). In the context of higher education, personal motivation and creativity, social capital (supportive social networks), and a knowledge-creating culture as well as access to information resources have been reported as important facilitation factors (16).

However, funding agencies are skeptical about the effectiveness and the added value of large cross-disciplinary initiatives when compared to less resource-intensive uni-disciplinary research (2). A discourse that may help to address these concerns is The Science of Team Science (TSTS), which has produced different approaches and models to assess and evaluate the functioning and effectiveness of cross-disciplinary teams. In their integrative literature review, Tigges et al. (17) provide an overview of existing teams science models and their measures of collaboration quality (e.g., team interactions and processes during collaboration) and collaboration outcomes (publications and citations). For instance, Stokols et al. (8) have suggested a conceptual framework to evaluate transdisciplinary research assessing large-scale scientific collaboration, scientific integration, health impacts, professional validation, and communication, differentiating between immediate, intermediate, and long-term markers. Wooten et al. (18) assessed team maturation and scientific progress in multidisciplinary teams using a mixed-methods design. Another approach to evaluate whether cross-disciplinary research initiatives effectively enhance research collaboration and long-term health impacts is Hall et al.'s (19) “Conceptual Model for Evaluation of Collaborative Initiatives.” It evaluates research collaboration processes and outcomes, differentiating between three phases of (a) collaborative readiness, (b) collaborative capacity, and (c) collaborative products.

This study reports on the E&C of science teams in Capital4Health (C4H), a transdisciplinary research consortium funded by the German Federal Ministry of Research (2015–2020), which aims to increase capabilities for active lifestyles across the lifespan. The consortium is based in Bavaria, Germany and is composed of research institutions as well as policy and professional partners. Following an interactive knowledge-to-action approach (20), partners seek to co-produce sustainable measures to promote physical activity in different settings. Settings include daycare centers, schools and universities, apprenticeship and vocational education, communities, and senior housing, and each are addressed by a dedicated sub-project.

The consortium has a specific focus on fostering E&C between the teams of scientists from five sub-projects, supporting them in jointly generating innovative solutions to the problem of physical inactivity and going beyond established discipline-specific approaches. Two cross-cutting projects support this process by providing theoretical input, supporting evaluation, and fostering consortium-wide interaction. Concrete interventions to foster E&C between projects have included expert workshops, annual meetings of the entire consortium with an international scientific advisory board, a network of all young researchers in C4H, semi-annual meetings of a transdisciplinary steering committee and all principal investigators (PIs) to coordinate consortium strategies, ongoing support by e-mail and phone, and regular group interviews with all project teams.

This study used a mixed-methods approach based on Hall et al.'s (19) three-stage model to analyze 5 years of E&C (2015–2020) in the C4H consortium. Given that (a) the capacity to cooperate needs to be developed during the collaboration process (21), that (b) collaborative outcomes are to be expected at the end of a project (8), and that (c) especially multi-university research projects benefit from intensive coordination efforts (22), we based our analysis on the following assumptions: (1) perceptions of E&C would increase over the project's life, (2) the number of collaborative products would increase toward the end of the project's life, and (3) the coordinating sub-project would qualify as an important support mechanism for E&C in this multi-university research consortium. From our perspective, gaining further insight regarding these assumptions and the temporal development of E&C during the lifetime of the project can provide insights into how to create conditions that are conducive to E&C (23) in health promotion in general and in physical activity promotion in particular. This might be of interest to both funding agencies and scientists embarking on cross-disciplinary collaborative projects.

## Methods

### Theoretical Framework

As mentioned above, we used Hall et al.'s (19) conceptual framework to guide our analysis. As suggested by the authors (19), we divided the project span into phases of collaborative readiness, collaborative capacity, and collaborative products.

*Collaborative readiness* refers to a broad range of circumstances that influence effective cooperation among teams in the initial project phase and that are crucial for a successful project (19, 24). Hall et al. (19) suggest assessing collaborative readiness through environmental, intra- and interpersonal measures including institutional resources or support (24), the local proximity of investigators (2, 19, 24), the diversity of scientific disciplines (24), and research orientation (19). Also, programmatic goal setting, the duration of the program, the research orientation of scientists and their leadership skills, the number of participating scientists (25), the presence of brokers (2), and researchers' prior collaboration on past projects have been identified as readiness factors (2, 24).

*Collaborative capacity* addresses the above-mentioned circumstances in the intermediate or later project phases (19). At this stage, factors such as team functioning and cognitive (e.g., a shared mental model), motivational (e.g., knowledge sharing), and behavioral team processes (e.g., trust, face-to-face communication) (2, 13) as well as credit for co-authorship, institutional work culture, and power relations (26) inevitably influence effective cooperation and exchange among researchers. Further, collaborative capacity is not a given for scientists but needs to be learned over time during the collaboration process (21).

Finally, *collaborative products* such as research, training or policy, and health outcomes are to be expected toward the end of a research project (19). The literature on research outcomes—also labeled knowledge outcomes (22)—shows that they are often assessed through co-authored publication metrics, conference or workshop presentations (22), or the social network analysis of co-authored publications (2, 13, 27–30). Chen (28) analyzes academic social networks (project-based, learning-based, and institution-based) and their respective collaboration mechanisms influencing cooperative research outcomes. Others (31, 32) have combined research team outcomes with data about research participants' perceptions, an approach that is also increasingly encouraged in the literature (27, 33–35). Co-produced publication metrics were criticized as merely a partial indicator of collaboration (34) as they tell little about cooperation dynamics and processes over time (19). Examples of training outcomes include successfully supervised graduate students' theses or dissertations (7) or industry jobs that graduate students have received (22). Policy and health outcomes refer to established collaborations with political or healthcare institutions (22), among others.

Based on empirical findings showing that publication metrics tend to underestimate collaborative processes and should be combined with the subjective views of collaborating researchers (31, 32), we complemented the original model with perceptions of E&C among researchers as well as with the number of collaborative products across all project phases (collaborative readiness, collaborative capacity, and collaborative products) to make the developmental and integrative processes of E&C visible.

### Data Collection

To collect data on these different aspects of E&C in the C4H consortium, we used a mixed-methods design based on (a) semi-structured individual interviews with the consortium's PIs at the start of the project (pre-interviews), (b) semi-structured reflexive focus-group interviews throughout the project's lifetime, and (c) document analysis of relevant project documents and scientific outcomes.

For the pre-interviews and the focus-group interviews, we developed interview guidelines (IG1, IG2) that were both compatible with the Hall framework but broad enough to allow for adaptation during different phases of the project (e.g., initial contacts with external partners in the early project stage or workshops on a potential new funding phase toward the end). In line with Hall et al. (19) and due to data availability, we chose the research orientation of PIs, local distances between sub-projects, and diversity of scientific perspectives as measures of collaborative readiness. Firstly, to assess research orientation, we analyzed qualitative telephone pre-interviews with the sub-project PIs conducted in 2016 by a former member of the coordinating project. The interview guideline (IG1) included questions on previous experience in cooperating with scientific partners (IG1_Q1), difficulties experienced during these cooperative endeavors (IG1_Q2), potential success factors (IG1_Q3), and personal motivation to participate in the research consortium (IG1_Q4). Five pre-interviews were conducted between May and June of 2016, lasting ~30–45 min each.

In addition, a total of 23 semi-structured focus-group interviews were conducted by the first author (SF) or her predecessors between 2015 and 2020, and all interviewers were adequately trained in interviewing techniques. Due to Covid-19 induced difficulties, the last planned focus-group interview in 2020 was transformed into a semi-structured individual interview as only one of the invited focus-group participants was able to participate. The interview guideline addressed questions on the current status of the sub-project (IG2_Q1), expectations regarding the further project work (IG2_Q2), cooperation with project partners (IG2_Q3), and cooperation within the research consortium (IG2_Q4). The interviews took the form of semi-structured reflexive focus-group interviews conducted with the scientific sub-project teams on a semi-annual and later on an annual basis either face-to-face at the lead University of the respective sub-projects or *via* web conference in 2020 (due to the COVID-19 pandemic). The number of focus-group participants varied over time and ranged from two to six researchers. Focus-group interviews lasted ~30–90 min each, the individual interview about 90 min.

All (pre- and focus-group) interviews were audio-recorded and transcribed. Identification elements (person names, places, institutions or factory names) were anonymized, and participants signed consent forms before participating in the interviews. Transcripts of all interviews were transferred to the qualitative analysis software MAXQDA (version 20). Final transcripts were not returned to participants for correction or comment, and participants were not asked to provide feedback on the findings. The interview quotes used in this article were translated from German to English by the first author and verified by the second author. This work adheres to the COREQ criteria for reporting qualitative results (36).

### Document Analysis

While researchers' subjective views on cooperation, as reflected in interviews, provide a grasp on the dynamics and processes of cooperation (19, 31), the analysis of research outputs, such as co-authored publications, is considered an objective measure of cooperation that can be applied validly and reliably across research settings (19). Although such products are expected toward the end of a project, we aimed at analyzing research products across all project phases. The first author collected the number of published scientific articles or books and both published or unpublished conference abstracts from a shared electronic storage folder used by all sub-projects and the common C4H website and sub-projects' University homepages. We further analyzed the number of training outcomes, such as finished bachelor's, master's, or doctoral theses supervised by researchers of the consortium. The first author sent a small survey *via* e-mail to all sub-projects, asking them to complete a list with all completed works (for bachelor's, master's, or doctoral theses) during the entire project of C4H (2015–2020). Additionally, and with regard to collaborative readiness measures, the C4H grant proposal was included and provided important basic information on the participating teams and disciplines.

### Data Analysis

We used content analysis (37) to explore the research orientation among the participating PIs as reported in the pre-interviews. This method involved a deductive definition of the main categories based on the interview guideline (IG1) and concepts of inter-, multi-, and transdisciplinary research. The first author (SF) repeatedly read all the interview material and generated codes and anchor examples based on the pre-defined categories. Subsequently, the second author (MT) reviewed the codes and categories. Finally, the category system was applied to the rest of the material. Discrepancies were discussed between SF and MT until consensus was reached.

We analyzed all interview questions (IG1_Q1 to IG1_Q4), collecting quotes indicating a PI's prior experience in cooperation with academic, practice, and policy partners. Inspired by Rosenfield (38), we considered a PI to be oriented toward inter-/multidisciplinarity if the researcher reported having had experience in cooperation with academic partners from other scientific disciplines prior to participation in C4H. Following Bergmann et al. (39), a transdisciplinary research orientation was assumed if the PI reported to have had experience in cooperation with academic, policy, and practice partners including the co-creation of solutions prior to participation in C4H. During the analysis, a third intermediate category was identified, namely experience in cooperation with policy and practice partners (research-practice-partnership orientation).

The focus-group interviews were first categorized into one of Hall et al.'s respective project phases based on the year in which they were conducted, as follows: collaborative readiness (interviews conducted in 2015–2016), collaborative capacity (interviews conducted in 2017–2019), and research outputs (interviews conducted in 2020). We used content analysis (37) of all interview questions (IG2_1 to IG2_3), performing an inductive coding approach to collect quotes illustrating perceptions of E&C among and between sub-projects. Quotes were extracted and coded by the first author (SF), who also classified the categories and sub-categories and developed a codebook. The second author (MT) revised the work, and divergences were discussed until agreement was reached.

The first author (SF) analyzed the grant proposal and extracted the University locations, university departments, and research areas of the research teams. She also compiled the numbers of published journal articles, books, degree-qualifying works, and conference abstracts according to the phase (collaborative readiness, collaborative capacity, research outputs) in which they were produced. The second author (MT) reviewed the analysis, and divergences were discussed until agreement was reached.

In the final step, data from all three data sources were compiled and, where possible, triangulated (40) to provide a comprehensive overview of E&C in the different project phases of C4H. Table 1 provides an overview of how evidence from the pre-interviews, the focus-group interviews, and the document analysis was used to inform the results reported below for collaborative readiness, collaborative capacity, and collaborative products, respectively. Data summaries were initially collated by the first author (SF), double-checked by the second author (MT), and finalized in discussions with the other co-authors.

**Table 1:** Mixed-methods design applied in this study.

**Data sources and methods**	**Project phases based on Hall et al**. **(****19****)**		
	**Collaborative readiness**	**Collaborative capacity**	**Collaborative products**
	**Measures**		
Qualitative content analysis of pre-interviews (*N* = 5; IG1) conducted in 2016 with five participating PIs (deductive approach)	Research orientation of PIs		
Qualitative content analysis of semi-structured reflexive focus-group/individual interviews (*N* = 23/*N* = 1; IG2) conducted between 2015 and 2020 with 5 sub-project research teams (inductive approach)	Perception of E&C among the sub-projects in 2015–2016	Perception of E&C among the sub-projects in 2017–2019	Perception of E&C among the sub-projects in 2020
Qualitative content analysis of grant proposal for the first funding phase (2013)	Diversity of scientific disciplines Local distance between sub-project teams		
Quantitative analysis (count) of the number of publications (scientific articles, books, conference presentation) retrieved from University websites, shared electronic storage, and the C4H-website	Number of publications in 2015–2016	Number of publications in 2017–2019	Number of publications in 2020
Quantitative analysis (count) of survey on the number of degree-qualifying works (bachelor's, master's, and doctoral)	Number of degree-qualifying works in 2015–2016	Number of degree-qualifying works in 2017–2019	Number of degree-qualifying works in 2020

### Participants

Participants consisted of members of the C4H project research teams, including PIs (males, *N* = 5; females, *N* = 3—all University professors) and research associates at the postdoctoral or early researcher levels. A consistent number of research associates and their genders and ranks cannot be ascertained due to staff fluctuation throughout the lifetime of the projects. A total of *N* = 5 sub-projects was included in the analysis. Since the coordinating sub-project conducted the reflexive interviews analyzed in this study, it was excluded from all analysis. Also, the evaluating sub-project was omitted because it did not take part in the reflexive interviews.

## Results

The qualitative content analysis of the pre-interviews resulted in *N* = 10 codings falling into the aforementioned main categories of “inter-, multidisciplinary research orientation,” “research-practice-partnership orientation,” and “transdisciplinary research orientation.” With regard to the analysis of the focus-group interviews, we identified a total of *N* = 152 codings. The identified main- and sub-categories are shown in Table 2. The main categories include “general perceptions of E&C among the sub-projects,” “perceived challenges to E&C among the sub-projects,” “perceived facilitators for E&C among the sub-projects,” “E&C with the coordinating project,” “E&C with the evaluating project,” and “E&C with the Young Researchers Network.” In 2020, additional categories addressing E&C among the PIs (see Table 2) were identified. In the following, the results of the analyzed interviews as well as of the collaborative products are presented according to the project phase in which the data were collected. Results dealing with E&C with the coordinating and evaluating sub-projects as well as with the Young Researchers Network are reported for the entire project phase to make reading more coherent. Quotations from the interviews are provided to illustrate the main findings. Participants are identified by research team and year.

**Table 2:** Category system for pre-interviews and semi-structured focus-group interviews.

**Project phase**		**Collaborative readiness**	**Collaborative capacity**	**Research outcomes**
**Interview**	**Main category**	**Sub-categories**	**Sub-categories**	**Sub-categories**
Pre-interviews with PIs	Research orientation (deductive categories)	- Multi-/interdisciplinary: experienced in cooperation with academic partners from other academic disciplines prior to the participation in C4H - Research-practice partnership: experienced in cooperation with policy and/or practice partners prior to the participation in C4H - Transdisciplinary: experienced in cooperation with academic, practice, and policy partners including the co-creation of new solutions prior to the participation in C4H (transdisciplinary)	-	-
Semi-structured focus-group interviews with sub-project research teams	General perception of E&C among the sub-projects (inductive categories)	- None to little	- None or little - Increasing	- None or little - Increasing - Exchange without co-production/cooperation
	Perceived challenges to E&C among sub-projects (inductive categories)	- Lack of trust - Start-up phase of the project - No perceived benefit of E&C - Own project load - No planned occasions of E&C - E&C as time-consuming development process - No clearly communicated goal of increased E&C - Too project-specific problems - Limited resources - Perceived reluctance for E&C among other sub-projects	- Focus on own project work - E&C have not been taken into account in project design from beginning on - Limited resources - Unpopular topics - Own project load - Too project-specific problems - E&C as add-on business - No clearly communicated goal of increased E&C - Staff discontinuities - Perceived reluctance for E&C among other sub-projects	- Own project load - Too project-specific problems - No perceived benefit - Limited resources - Unclear roles (who is defining tasks and who is pursuing them?) - Perceived reluctance for E&C among other sub-projects
	Perceived facilitators for E&C among sub-projects (inductive categories)	- Involvement of project members in two sub-projects - Young Researchers Network - Coordinating project - Events (workshops, advisory board meeting) - Shared problems	- Shared problems - Shared interests - Coordinating project - Events (workshops, advisory board meeting) - Local proximity - Experience of prior collaboration - Young Researchers Network	- Perceived benefit - Shared interests - Events (advisory board meeting) - E&C as mandatory task - Young Researchers Network - Coordinating project
	General perception of E&C among PIs (inductive categories)			- Increasing - Valued (helpful, positive, feeling of connectedness)
	Perceived facilitators for E&C among PIs (inductive categories)			- Shared goals (third funding phase, publication) - Clear structure and time frame - Coordinating project
**Interview**	**Main category**	**Sub-categories**	**Sub-categories**	**Sub-categories**
	E&C between a sub-project and the coordinating project (inductive categories)	- Coordinating project as the only E&C partner - Coordinating project as contact person	- Coordination and evaluating project as most frequent E&C partners - Coordination project perceived as exchange facilitator - Coordination project perceived as expertise holder/driver - Facilitators for E&C: local proximity, structure and transparency, enough resources - Work for the coordinating project perceived as add-on business - Valued (helpful)	- Valued (central role of the project) - Coordinating project as most frequent E&C partner - Facilitators for E&C: structure and transparency, enough resources
	E&C between a sub-project and the evaluating project (inductive categories)	- Regular contact with evaluating project - Valued contact (add-on business; positive)	- Evaluating and coordination project as most frequent E&C partners - Valued contact (positive, helpful)	- Valued contact (positive)
	E&C among sub-projects in the context of the Young Researchers Network (inductive categories)	- Young Researchers Network as a safe discussion forum for junior researchers	- Driven by engaged person of Young Researchers Network - Shared goal (special issue)	- Shared goal (special issue) - Scheduled, regular exchange

### Phase of Collaborative Readiness (2015–2016)

#### Research Orientation

Concerning their research orientation, all five PIs reported an inter-, multidisciplinary research orientation [experience in cooperation with academic partners from other scientific disciplines (*N* = 5)] and a research-practice-partnership orientation [experience in cooperation with practice and/or policy partners (*N* = 5)]. None of the interviewed PIs reported to have experience in cooperation with academic, practice, and policy partners including the co-production of new solutions (transdisciplinary research orientation).

### Local Distance Between the Sub-projects

Research teams were located in seven different cities in Germany, with six of them in the State of Bavaria and one in Hesse. Three sub-projects were located within the same University department, and one PI was involved in two sub-projects at different sites. In sum, distances of maximally 324 km were to overcome for meetings, and the time zone was the same for all involved sub-projects.

### Diversity Between Scientific Perspectives

The majority of researchers were affiliated with departments of sport sciences. However, specializations differed, including sports medicine (*N* = 1), physical education (*N* = 2), sport/rehabilitation science (*N* = 1), and public health and physical activity (*N* = 2). Other researchers were from the fields of health sciences (*N* = 1) and medical sociology (*N* = 1).

### Perceptions of E&C During the Collaborative Readiness Phase (2015–2016)

At the start of the project (2015–2016), the majority of participants reported perceiving no or little cooperation with sub-projects other than the coordinating or evaluating project, as illustrated by the following quote: “[…]but we actually do not notice anything [regarding any of] the other projects” (Participant, team 1; 2015).

One reason for little E&C was that the fulfillment of their own project tasks required all the resources of the sub-projects, especially during the start-up phase of the project. Also mentioned were lack of trust between researchers, the fact that E&C amid teams is a process that takes time to develop, and that proceeding in a structured way toward the clearly formulated goal of “E&C” was necessary. It was reported that researchers could not see a benefit to more aspects of E&C as becoming acquainted and building trust were more important at this stage of the project. Some teams were able to learn about other sub-projects through a cooperator involved in two different sub-projects. Generally, if E&C took place, it was facilitated through the coordinating project or the Young Researchers Network, which organized occasions to meet (e.g., workshops and advisory board meetings).

### Number of Research and Training Outputs

At this project stage, a total of 23 research outcomes was identified, including four conference presentations in 2015, and 17 conferences presentations in 2016. With regard to training outcomes, two bachelor's theses were supervised in 2016 (see Table 3).

**Table 3:** Co-produced publications, degree-qualifying works, and conference presentations between 2015 and 2020.

**Year**	**Publications**	**Mentoring of degree-required works**			**Conference presentations**	**Total outcomes**
		**Bachelor's**	**Master's**	**Ph.D**.		
2015					4	**4**
2016		2			17	**19**
2017	3		2		12	**17**
2018	1	1	1		13	**16**
2019	4	1	2	1	23	**31**
2020	11		1	1	1	**14**
**Total**	**19**	**4**	**6**	**2**	**70**	**101**

### Phase of Collaborative Capacity (2017–2019)

#### Perceptions of E&C During the Collaborative Capacity Phase (2017–2019)

The reports of the participants suggested a tendency toward increased E&C among sub-projects compared to the beginning phase. Among the facilitators mentioned for E&C were local proximity or prior experience working together. Several participants described shared interests or problems as additional facilitators. However, the E&C did not go beyond exchange or lead to the co-creation of new materials, as indicated in the following words from an interview:

“[…]Team 1 and team 5 are two sub-projects dealing with health literacy and…we are basically working [on parallel tracks] and have developed independently of each other the constructs of physical-activity-related health literacy (team 1) and sport-related health literacy (team 5)…Every now and then we talk about what is similar or perhaps different, but we work mostly in [a parallel way] […]” (Participant, team 5; 2019).

A major challenge to promoting E&C was limited resources. Researchers appreciated learning about new approaches through interdisciplinary exchange with other sub-projects but perceived E&C as an “add-on” business. Further, the project design of putting sub-projects in diverse contexts and defining topics and goals for every sub-project was perceived as a barrier that prevented increased E&C as the sub-projects were primarily consumed by their own workloads. E&C was not considered a source of added value for the work of individual projects since the settings were too different.

### Number of Research and Trainings Outputs

At the collaborative capacity stage, a total of 48 research and training outcomes was produced. In 2017, three scientific publications, two master theses, and 12 conference presentations (a total of *N* = 17 products) were counted. In 2019, the total number of research and training products increased to 31 with four scientific publications, 23 conference presentations, and four degree-qualifying works (bachelor's, master's, and Ph.D.) (see Table 3).

### Phase of Collaborative Products (2020)

#### Perceptions of E&C During Collaborative Products Phase (2020)

Toward the end of the project (2020), E&C was reported to occur in the form of several joint activities—the PIs worked collaboratively on a publication about the research consortium, they met to discuss options to apply for a third funding phase, and the young researchers jointly worked on the publication of a special issue for a relevant journal in the field of public health. The meetings of the PIs for the joint publication and discussions about ideas for a potential third funding phase were observed as highly positive and created feelings of connectedness with the consortium, a spirit of optimism, and motivation for closer E&C among the participants of the consortium. Through work on the publication, PIs discovered commonalities among the sub-projects, a common goal (the publication), the resulting benefits of the undertaking (e.g., producing the publication), and the increased visibility of the consortium. One researcher stated,

“[…] we made a shared outline for the publication and realized that it is all quite fitting. There are many parallels between the projects…Somehow we are a consortium that is [having] similar experiences regarding similar topics, [sharing] similar ideas and perspectives.” (Participant, team 2; 2020)

Also, the mandatory nature of E&C to produce the publication and the clear structure and time frame with regard to the publication were perceived as facilitating E&C. As evidenced here, E&C with the coordinating project and the Young Researchers Network, both pushing forward the expansion of publications in scientific journals, was seen as a benefit.

In addition, researchers still perceived the E&C among sub-projects to be facilitated through events organized by the coordinating project or the Young Researchers Network. However, problems of high workloads of the sub-projects, limited resources, staff discontinuities, and the assumed reluctance of other sub-projects to engage in E&C continued to be reported at this project stage.

##### Number of Research and Training Outputs

The number of conference presentations was low (*N* = 1) in 2020 due to the COVID-19 pandemic. However, the highest number of scientific publications (*N* = 11) was produced during this year (see Table 3). A number of two supervised training outcomes (master's and doctoral theses) was reported.

### E&C Between the Sub-projects and the Coordinating Sub-project, the Evaluating Sub-project, and the Young Researchers Network

Across all three project phases, respondents addressed the E&C with the coordinating and the evaluating sub-projects as well as with the Young Research Network. The results are summarized in the following.

#### a) E&C Between a Sub-project and the Coordinating Sub-project

The coordinating project was determined to be the major contributor to E&C for all sub-projects across the time frame of the entire project. One participant described its central role as facilitator for the connection and the collective exchange between the sub-projects with the words

“[…] imagining it as a picture, …[the coordinating project] would perhaps be at the center surrounded by the sub-projects, each having a connection to [the coordinating project], and through [the coordinating project] we might have a meeting with each other” (Participant, team 2; 2015).

Moreover, the participation at events such as workshops and the advisory board meeting organized through the coordinating sub-project was perceived as a major enabling factor for E&C between the sub-projects. The coordinating sub-project was seen as an expert on theoretical concepts and therefore considered responsible for identifying the needs of the sub-projects and providing input on different relevant topics. While the project design of the first funding phase was criticized for not having transparently communicated that the sub-projects themselves would be the objects of the research of the coordinating sub-project, additional resources for cooperation in the second funding phase as well as more transparent communication by the coordinating project significantly improved the collaboration between the sub-projects and the coordinating project.

#### b) E&C Between a Sub-project and the Evaluating Sub-project

Following E&C with the coordinating project, E&C with the evaluating project was also conveyed frequently over the course of the project. It was understood by the participants to be particularly helpful, according to the following reflection:

“That was a good and regular exchange. We sat down together. [The scientist from the evaluating project] once even joined us in a residential home. That was excellent. Because you got to know each other and she also saw…the setting. I had the feeling she was more able to complete [the protocols] than us. After that, we learned how to do it and we did it as [well]. That is why the exchange with [the evaluating sub-project] was very good.” (Participant, team 4; 2018)

While researchers originally considered the tasks to be performed for the evaluating project to be inconvenient or extra work, they eventually found them to be useful for their own project work.

#### c) E&C Among Sub-projects in the Context of the Young Researchers Network

From the onset of the project, the Young Researchers Network was reported to be an important establishment, enabling exchange among novice researchers. Through workshops and common work on a special issue, the young scientists involved saw the Young Researchers Network on one hand as an organized structure for E&C and on the other hand as a space to express thoughts in a smaller setting. One such researcher posited,

“I think it is a good structure, …to try to get all junior researchers of all projects at one table…we all benefit from that” (Participant, team 5; 2020).

## Discussion

Given the criticism by funding agencies concerning the effectiveness of long-lasting and resource-consuming transdisciplinary research initiatives (2, 41), this study contributes some vital aspects to a better understanding of the development of cooperative processes and products among scientific teams in a physical-activity-promotion research consortium. Based on Hall et al. (19), we separated the study period into the phases of “collaborative readiness (2015–2016),” “collaborative capacity (2017–2019),” and “collaborative products (2020).” Factors of cooperative readiness were assessed and research and training outcomes as well as perceptions of E&C reported by the participating researchers themselves were analyzed for each project phase. Facilitators of and challenges to effective cooperation during the respective project phases were identified.

With regard to the assessment of researchers' collaborative readiness, all participating PIs reported having had prior experience in cooperative work but not in transdisciplinary cooperation. These results indicate a research orientation inclined toward scientific collaboration among all researchers (19). Moreover, all sub-projects except one were located within one German state and some of them even within the same University department. This local proximity may have fostered collaborative readiness due to the fact that intramural and domestic collaboration (42) as well as frequent face-to-face communication enhance research productivity. In addition, the reported scientific disciplines varied, and further research is needed to assess whether they were different enough to produce innovations and suppress groupthink (43). Our analysis showed that lack of trust, limited resources, lack of a clearly communicated goal of E&C, and the perceived reluctance for E&C among the other sub-projects were challenges in the early project phase, thus confirming previous results from the literature (2, 13, 23, 25).

The following finding resulting from the combined analysis of readiness measures and focus-group interviews merits highlighting: Although all analyzed aspects seemed to speak in favor of collaborative readiness among researchers from an outside-perspective, the researchers themselves reported to perceive little or no E&C among the sub-projects.

Later, during the phase of collaborative capacity, the awareness of E&C grew considerably, and although the co-creation of scientific knowledge had not yet been achieved, common interests and problems were identified. Perceived problems were, besides heavy individual workloads, limited resources, the complex project design, and diverse intervention settings as well as the perceived reluctance for E&C among the other sub-projects. Finally, by the end of the project (phase of collaborative products), the researchers reported increased E&C and perceived a benefit of E&C although they experienced exchange rather than cooperation. Both the PIs and the young researchers experienced the collaboration on joint scientific publications as an occasion for regular exchange and increasing identification of commonalities and a sense of unity.

In sum, our first assumption of increasing E&C over time could be verified by these results. Although the perceptions of E&C increased over time, the specific challenges to E&C identified in 2015/2016 were also reported in 2017/2018 and in 2020, specifically, limited resources, one's own project load, perceived reluctance among other sub-projects, and the diversity of settings. This supports the findings from the literature indicating that the level of collaborative readiness influences the success of the entire project (14, 19).

We found an increasing number of scientific publications and conference presentations over time, a finding that is in line with the literature (9) and confirms our second assumption. The co-production of publications was first reported in 2020. Several degree-qualifying works were supervised by the research consortium, and although mentoring has been reported as a motivating factor for collaboration (44), the researchers did not report having cooperated in the supervision of those theses. These findings confirm the literature suggesting that the number of publications (and research outputs more generally) provides limited information on the actual grade of cooperation (34) and even less on the knowledge integration among researchers (45). In sum, E&C in the research consortium seemed to be perceived as increasingly positive by the researchers toward the end of the project phase. Additionally, increased numbers of collaborative products coincided with perceptions of increased exchange even though the co-production of new integrated knowledge had not yet been achieved.

Concerning our third assumption, we found evidence that the coordinating project was a major cooperation partner and supporting factor for E&C among the sub-projects. Providing expertise on theoretical issues and more importantly organizing events to foster effective E&C among researchers, it was thought to provide essential support. This is confirmed by Cummings and Kiesler (22), who found a negative relationship between multi-university collaboration and project outcomes, which could be explained by insufficient coordination. Our findings further underscore the importance of a coordination unit that ensures structured exchange and organizes events for the enhancement of E&C in complex research initiatives. Also, other groups established by the research consortium (e.g., the Young Researchers Network and the evaluation project) proved to be useful for E&C once their potential benefits were recognized by the research teams.

Despite the inherent advantages of Hall et al.'s (19) model, it neither allowed us to assess the iterative collaborative processes necessary to achieve new stages of collaborative capacity nor to differentiate between different project goals emerging at different points in time. For this purpose, future research might benefit from using the four-phase model of Transdisciplinary Research (46), which includes iterative pathways and accounts for differences in goal setting, team types, and key processes across different project phases. Moreover, the project design of the C4H consortium is highly complex due to the diversity of its intervention settings. To adequately address this, future analyses of research consortia could greatly benefit from multi-team systems research (47). This approach conceptualizes science teams as networks pursuing shared superordinate goals in addition to their own team goals (48) and distinguishes between within- and between-team processes and properties at different levels of analysis (47).

Returning to the criticism by funding agencies concerning consortium-based work, this study indeed found evidence for their long-lasting and resource-consuming nature. However, the findings might provide helpful information for funding agencies and researchers writing grant proposals for cross-disciplinary research initiatives.

The most important study finding is the discrepancy between the assessment of collaborative readiness resulting from the objective analysis and from the focus-group interviews. While local distances and the diversity of disciplines and research orientation are important readiness factors, the perceptions of researchers showed that their needs for consortium-based E&C (e.g., sufficient resources, lower project loads of sub-projects, less complex project design, and clearly communicated goals of increased E&C) were different and remained a topic during the entire project life. Given the long list of threats to and facilitators of collaborative readiness (14, 49), this warrants the question as to which factors should be assessed and evaluated before a project starts.

Further, our results showed that the researchers indeed learned to exchange and collaborate over time. The reported development of increasingly positive attitudes toward E&C and the onset of co-produced products at the final stage suggest that personal, temporal, and financial investments into the research consortium seem to pay off. Particularly, overarching organs such as the coordinating project or the Young Researchers Network proved to play indispensable roles in the promotion of E&C and need to be equipped with sufficient resources. After 5 years of consortium-based work, the researchers seemed to have acquired capabilities for effective and successful cooperation. If long-term research funding was provided, these conditions might be an optimal starting point to achieve knowledge-integration and future health impact over the long run.

Participatory research generally refers to the involvement of non-academic stakeholders, such as community members or partners from civil society and policy, into the research process (50, 51). While this study concentrated on E&C among interdisciplinary researchers, our results mirror existing findings on participatory processes in researcher–stakeholder partnerships (51): Both the capacity of E&C and mutual understanding increased over time, while continuing efforts were needed to establish and maintain effective partnerships and mutual trust between all participating actors. Additionally, our results show that diverse study contexts, varying priorities or conflicting interests among researchers challenged participatory processes. Similar results have been observed by Roura et al. (52) among different academic and non-academic stakeholders. Other factors seem to be more specific to the academic context, such as the pressure to publish study results, the need to apply for new funding sources and frequent staff fluctuation. Also, power dynamics have been identified as an important influence factor in participatory research (52), which might be of particular interest in the highly competitive academic setting in future studies. In sum, and restricted to the academic context, our results suggest that there are both general and academia-specific factors that will influence the success of participatory and co-creative processes within research consortia. They need to be considered from the stage of project design onward—by researchers and funding agencies alike.

There are several limitations of this study. Firstly, our study results do not contribute completely new knowledge to the field of team science and readiness. However, certain findings merit highlighting as we have discussed above. Secondly, the study results are only applicable to the specific context of the C4H consortium. Research initiatives in different contexts might require different evaluation approaches and measures. Also, the sample size was relatively low, and conclusions need to be drawn with caution and complemented by further research. Further, the analysis of the included journal publications was not based on a systematic search on the Web of Science as not all articles could be found there (e.g., German publications not registered in the international databases). Including such an analysis would have made it possible to consider questions of research impact and the number of citations of sub-projects within other sub-projects. Moreover, the analysis of journal article authorship is not an optimal measure of research co-production. Further research should combine qualitative data with additional, potentially more valid and reliable measures of scientific co-production. For instance, a network analysis of co-authored publications could supplement the empirical evidence on communication modes and frequencies. Due to staff fluctuations, we did not differentiate our analysis according to gender although research shows sex-related differences with regard to cooperation strategies (53) and impact of scientific outcomes (27). Lastly, the combination of qualitative and quantitative assessments of outcomes has the advantage of identifying the processes influencing scientific co-produced outputs, but the lack of reproducibility and problems of intrusion cannot be ignored (45).

## Conclusion

This study analyzed the development of perceptions of E&C and of collaborative products in a consortium for physical activity promotion over an entire project cycle (5 years). The results show that a participatory research approach taking into account the perspectives on and requirements for scientific E&C even right from the stage of the project design might lay the ground for suitable, supporting, and transparent conditions for effective and successful E&C. Moreover, the unique individual and contextual conditions of each research initiative need to be considered, and it might be misleading to suggest one approach to measure collaborative readiness adaptable across contexts. This may help prevent or shorten long-lasting processes during which teams perceive E&C as challenging add-on business due to complex project designs, restricted resources, and unclear goals. To achieve the production of new solutions for pressing societal issues such as physical inactivity, time and personal efforts need to be invested to support researchers in their development of capabilities for E&C. Additionally, adequate financial and long-term funding of researchers including the establishment of a coordinating organ responsible for scanning researchers' needs and providing them with relevant information and E&C supportive skills are indispensable. Although cross-disciplinary research consortia are resource demanding, our results show that investments are important to support researchers during their learning process toward the co-production of new knowledge and societal impact.

## Data Availability Statement

To protect the confidentiality of the participants, audio data will not be made available. Other requests to access the data should be directed to the corresponding author.

## Ethics Statement

The studies involving human participants were reviewed and approved by Ethical approval for research within CAPCOM was granted by the Ethical Committee of the Friedrich-Alexander University Erlangen-Nuremberg. The patients/participants provided their written informed consent to participate in this study.

## Author Contributions

SF collected, analyzed, and interpreted the data and drafted the manuscript. MT participated in the interpretation of the data, in the translation of quotes and interview guides, and helped in the drafting and revision of the manuscript. KA-O, KP, and PG critically reviewed and revised the manuscript. All authors read and approved the final manuscript.

## Conflict of Interest

The authors declare that the research was conducted in the absence of any commercial or financial relationships that could be construed as a potential conflict of interest.
